# Diffusion-weighted magnetic resonance imaging as a promising technique for detection and follow-up of liver metastases from neuroendocrine tumors: a retrospective and prospective study from the NETTARE task force

**DOI:** 10.1007/s40618-026-02974-5

**Published:** 2026-08-14

**Authors:** Tiziana Feola, Franz Sesti, Alessia Filice, Alexandra Nistor, Alessandra Valenti, Giulia Puliani, Roberta Centello, Carla Pandozzi, Arianna Gagliardi, Riccardo Pirisino, Oreste Bagni, Mauro Salducci, Marialuisa Appetecchia, Carlo Catalano, Andrea M. Isidori, Francesca Maccioni, Elisa Giannetta

**Affiliations:** 1 Dept. of Experimental Medicine, Sapienza University of Rome, Rome, Italy; 2 Neuroendocrinology, Neuromed Institute, IRCCS, Pozzilli, Italy; 3 Department of Wellbeing, Health and Environmental Sustainability - BeSSA, Sapienza University of Rome, Rieti, Italy; 4 Department of Radiological, Pathological and Oncological Sciences, Sapienza University of Rome, Rome, Italy; 5 Oncological Endocrinology Unit, IRCCS Regina Elena National Cancer Institute, Rome, Italy; 6 Department of Nuclear Medicine, Santa Maria Goretti Hospital, AUSL Latina, Latina, Italy; 7 Department of Sense Organs, Sapienza University of Rome, Rome, Italy; 8 Policlinico Umberto I, Centre for Rare Diseases (Endo-ERN Accredited), Rome, Italy

**Keywords:** Neuroendocrine tumors, Neuroendocrine neoplasms, Liver metastases, Magnetic resonance, Diffusion-weighted magnetic resonance

## Abstract

**Background:**

A combination of morphological and functional imaging plays a crucial role for neuroendocrine tumors (NETs) management. Diffusion-weighted magnetic resonance imaging (DWI-MRI) is an emerging key imaging technique in the NET field.

**Aim:**

to evaluate the diagnostic and prognostic role of DWI-MRI in liver metastases (LMs) from NETs compared with multiparametric MRI and functional imaging with ^68^Ga-DOTA peptides positron emission tomography (PET)-computed tomography (CT).

**Methods:**

This is a monocentric, observational, both retrospective and prospective study including patients with histologically proven diagnosis of gastroenteropancreatic (GEP) NET with LM evaluated with MRI and ^68^Ga-DOTA peptides PET-CT, performed within 6 months apart at diagnosis or follow-up. We collected clinical, pathological, imaging data including lesions number detected on (a)MRI-gold standard, (b)DWI images, (c)^68^Ga-DOTA peptides PET-CT. Apparent diffusion coefficient (ADC) was calculated on target lesions.

**Results:**

426 LMs were analyzed from 17 patients (11 males, mean age 57.2 ± 12.6 years) meeting the inclusion criteria (11.8% G1, 64.7% G2, and 23.5% G3); MRI detected a median of 15 (IQR 2.5–40) lesions per patient, DWI 15 (IQR 2.5–36.5) lesions, while PET-CT 6 (IQR 2.5–20) lesions. Non-parametric analyses suggested that DWI had a detection rate comparable to MRI (*p* = 0.068) and superior to 68Ga-DOTA peptides PET-CT (*p* = 0.004), whereas mixed-effects modelling accounting for within-patient clustering showed no statistically significant pairwise differences between imaging modalities after multiple-comparison correction. Moderate-to-good consistency in lesion burden estimation across imaging modalities supported the reproducibility of lesion quantification. Correlation analyses suggested MRI lesion load may reflect the aggressiveness of disease.

**Conclusions:**

DWI-MRI can be a useful time-saving imaging technique in the diagnosis and follow-up of LMs from NETs, with a potentially favorable cost-effectiveness ratio.

## Introduction

Neuroendocrine Neoplasms (NENs) are a heterogeneous group of malignancies in terms of origin, localization, and clinical presentation. This heterogeneity, together with the often-subtle nature of symptoms, frequently leads to a diagnosis only when patients already present with liver metastases (LMs). Different conventional - computed tomography (CT), magnetic resonance (MR)- and functional - positron emission tomography (PET) - imaging methods are essential for correct diagnosis, staging, assessment of treatment response, and follow-up of NENs [1, 2]. MR imaging (MRI) has some advantages over CT such as the absence of ionizing radiation, the non-use of iodinated contrast medium, the superiority in the analysis of soft tissues and non-hypervascularized lesions and a lower inter-observer discordance. Furthermore, MRI is more adequate than CT for the study of small lesions and has higher resolution for pancreatic NENs (pNENs) and LMs [3–5].

In the last years, diffusion-weighted imaging (DWI)-MRI has been emerging as a key tool in tumour detection, characterization, and monitoring and predicting treatment response [6]. DWI is a functional MR technique that allows quantitative evaluation of water proton diffusion in tissues. The restriction of water diffusion can be then quantitatively analysed with the calculation of the apparent diffusion coefficient (ADC). In general, the ADC value is inversely proportional to cellular density of a mass and tumours with high cellularity and densely packed cells tend to exhibit relatively restricted diffusion [7]. DWI is commonly used in several clinical scenarios, besides still being a hot research topic: it has been tested in almost all cancers to differentiate malignant from benign lesions, to distinguish different malignant histotypes or tumour grades, to predict and/or assess treatment responses, and to identify residual or recurrent tumours in follow-up examinations [8–10].

The widespread use of this new non-invasive technique in oncological imaging has led to a wide number of studies and promising data towards the introduction of this sequence in routine MRI protocols for several tumours, including NENs [11, 12]. DWI showed an accurate detection rate of LMs from advanced gastroenteropancreatic (GEP) neuroendocrine tumours (NETs) [13] and of pNETs in multiple endocrine neoplasia (MEN)−1 patients [14] as compared to standard MRI approaches. Notably, DWI-MRI demonstrated comparable results to ^68^Ga-DOTANOC PET-CT in pNETs patients [15]. Additionally, DWI-MRI has been identified as an important tool in predicting NEN grade, suggesting that ADC value obtained with DWI-MRI, could represent a non-invasive and valuable imaging parameter in predicting the WHO grade of tumours in pNET [16–20].

These recent investigations suggested DWI-MRI as a possible modality to stage patients and to assess treatment response, with peculiar advantages: (1) a quick, easily replicable sequence, more available than PET-CT; (2) a safe technique, radiation-free, for patients with favourable prognosis and long-term follow-up. In this context, we proposed a monocentric, observational study, both retrospective and prospective, aimed to explore the diagnostic performance of DWI sequences in comparison with multiparametric (mp)MRI and Ga-DOTA peptide PET-CT in the detection and characterization of LMs from NETs, as well as their potential prognostic correlation with tumour proliferation and disease progression under somatostatin analogues (SSAs).

## Methods

### Study design and study population

This is a monocentric, observational study, including a retrospective and a prospective phase, on adult patients of both sexes with histologically proven NET associated with LMs that according to routine clinical practice and guidelines, underwent mpMRI and ^68^Ga-DOTA peptides PET-CT imaging within 6 months of each other at diagnosis or during follow-up. We excluded patients with MRI images and/or ^68^Ga-DOTA peptides PET-CT of unsatisfactory quality and with significant artifacts.

**Fig. 1:**
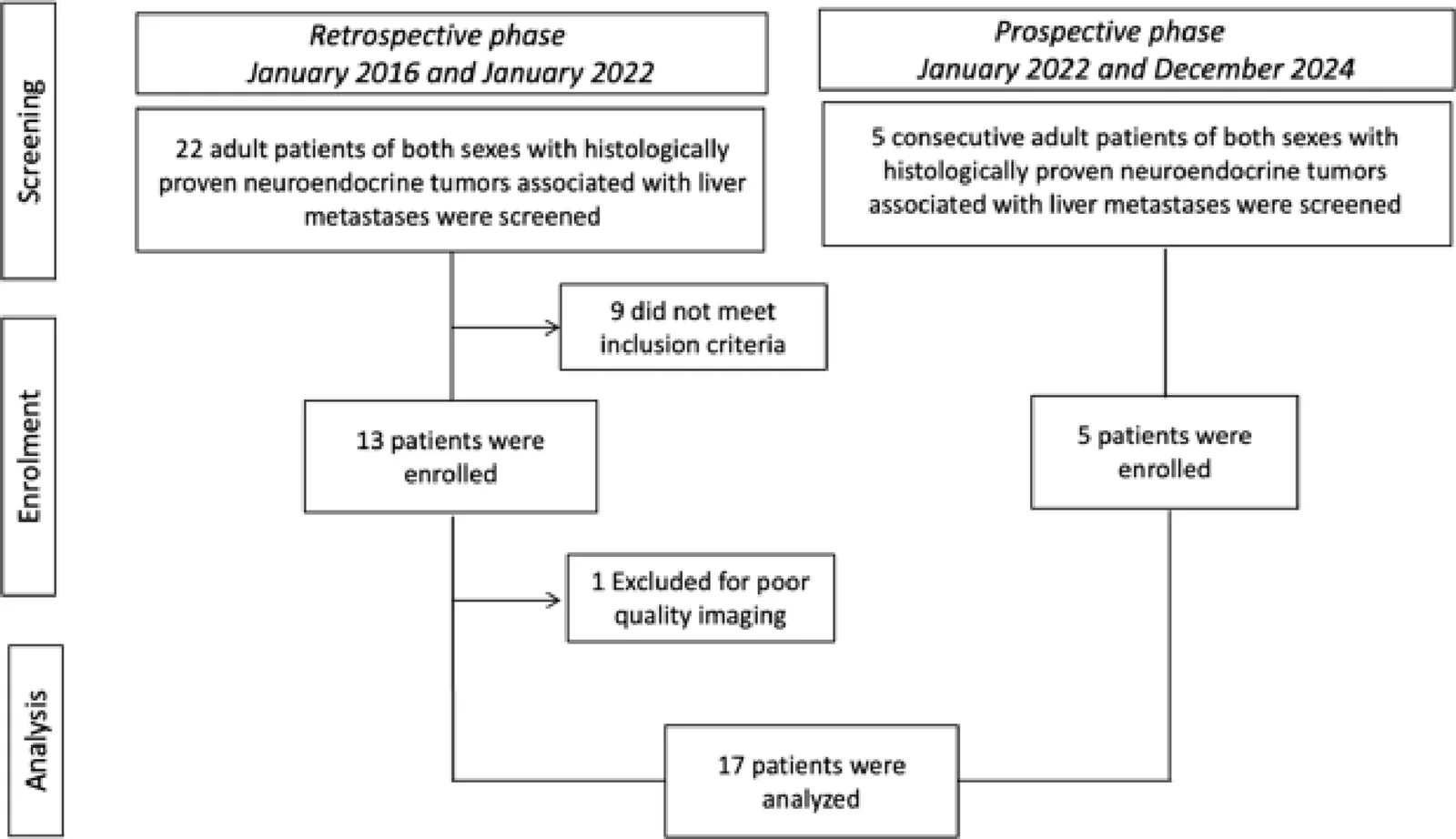
Study flow-chart

Through a retrospective review of medical records and electronic databases covering the period from January 2016 to January 2022, a total of 22 patients followed by the Neuroendocrine Tumour task force (NETTARE) multidisciplinary unit of “Sapienza” University of Rome–Policlinico Umberto I- with a histological diagnosis of NET and LMs were selected. Of these, 13 met the inclusion criteria, but 9 patients were excluded due to the absence of the DWI sequence, and an additional 1 was excluded due to poor image quality or the presence of artifacts. Among patients evaluated prospectively at the NETTARE Unit between February 2022 and December 2024, 5 patients met the inclusion criteria and were therefore enrolled. Figure 1 shows a flowchart summarizing the enrolment process.

We collected the following data: sex, age at diagnosis, clinical symptoms and signs; NET site, grading, staging, treatment and status at follow-up (stable disease or progressive disease).

The study was approved by the Ethical Committee of Policlinico Umberto I (ref. number 6780) and performed in accordance with the ethical standards as laid down in the 1964 Declaration of Helsinki and its later amendments. All patients provided written informed consent after the purpose and the nature of all procedures had been fully explained. This study adhered to the Strengthening the Reporting of Observational Studies in Epidemiology (STROBE) guidelines for reporting.

### Sample size

We based our hypothesis on a previous study by C. Schraml et al. [21]. A sample size of 145 lesions achieves 80% statistical power to detect a difference of −0.070 between DWI-MRI and PET-CT techniques whose sensitivities are 0.990 and 0.920, respectively. The calculation was based on a two-sided McNemar test with a significance level of 0.05, assuming a disease prevalence of 1.000 and a proportion of discordant pairs of 0.088. Although the final inferential analyses incorporated clustered lesion counts through mixed-effects models, sample size estimation was based on a simpler lesion-level paired detection framework using McNemar assumptions, consistent with previously published imaging studies.

### Imaging analysis

All MRI examinations were performed on a 1.5-T system with multiple phased-array surface coils and receiver channels using an integrated parallel acquisition technique (Avanto, Siemens Healthcare, Erlangen, Germany). Two experienced radiologists (F.M and C.C) reviewed independently the number and location of the liver lesions detected by complete mpMRI, in T2 and T1 sequences enhanced with gadolinium (arterial, portal and late phases), in the hepatobiliary phase and in DWI. In the case of many lesions (> 20), the number of lesions in the left hepatic lobe only was recorded to ensure methodological consistency and feasibility of systematic lesion-level evaluation.

The same lesion identification and counting strategy was applied by experienced radiologists in a consensus approach using predefined criteria, with discrepancies resolved by joint review. For each patient, up to three target lesions were selected, identifying the largest and most solid ones. The restriction to up to three target lesions per patient was introduced to ensure standardization of measurements (e.g., ADC and SUV) while limiting intra-patient correlation and measurement burden. The ADC of each lesion was calculated on the DWI images, using a T2-weighted morphological sequence as an anatomical reference, placing a circular or elliptical region of interest (ROI) on a homogeneous portion of each lesion, without involving the tissue interface neighboring areas and avoiding necrotic areas or degradation of moving artifacts. b values of 100, 800 and 1000 s/mm2 were used.

The ^68^Ga-DOTATOC PET-CT images were imported into the PACS (Picture Archiving and Communication System) of Policlinico Umberto I and transferred to the Nuclear Medicine UOC of the Santa Maria Goretti Hospital in Latina, part of the NETTARE Unit. Two nuclear medicine specialists (R.P and O.B) analyzed independently the images and recorded the number of LMs detected on PET-CT. For each of the target lesions identified on MRI, they also calculated the standardized uptake value (SUV), a semiquantitative measure of radioactivity concentration within a region of interest (ROI), normalized to the patient’s body weight.

As a histopathology-based gold standard was not realizable for every lesion detected, the mpMRI was established as a surrogate standard of reference [22]. In cases where there was a discrepancy between the number of lesions identified on DWI and those detected on PET-CT, the DWI findings were assessed in conjunction with the full mpMRI dataset. If the lesions observed on DWI exhibited the same imaging characteristics across the other mpMRI sequences—and were consistent with those confirmed by functional imaging and with all available clinical information—we concluded that these lesions were also secondary to NETs.

### Statistical analysis

Descriptive statistics were reported as means and standard deviations (SD) or medians and interquartile ranges (IQR), as appropriate to data distribution. Given the small sample size and the non-Gaussian distribution of most variables, non-parametric methods were prioritized. Normality was assessed using the Shapiro–Wilk test for descriptive purposes. Comparisons of lesion counts detected by mpMRI, DWI, and PET-CT in the same patients were first examined using Friedman’s test, as a non-parametric alternative to repeated-measures ANOVA. Pairwise comparisons were then made using Wilcoxon signed-rank tests with Bonferroni correction for multiple testing. These analyses were conducted without considering between-patient variability for lesion counts in each patient. Because lesion counts are intrinsically nested within patients, lesion-level analyses were interpreted primarily as measures of imaging detection burden rather than independent diagnostic events. However, considering the hierarchical nature of data, we then proceeded to use a complementary approach to account for within-patient clustering, analysing lesion counts of each imaging modality using generalized linear mixed-effect models (GLMMs). Imaging modality was included as a fixed effect, while a random intercept for patient was used to account for between-patient variability in lesion counts. The model followed a Poisson distribution with a log link function. To evaluate the presence of overdispersion, an alternative mixed-effects model assuming a negative binomial distribution was also fitted and compared with the Poisson model using information criteria, Akaike and Bayesian Information Criteria (AIC and BIC, respectively). Marginal means for pairwise comparisons of imaging modalities were then estimated after adjusting for sequential Bonferroni correction for multiple comparisons. Consistency of lesion counts across imaging modalities was explored descriptively using the Intraclass Correlation Coefficient (ICC), without implying diagnostic agreement. Pearson correlation coefficients were calculated to examine correlations between the number of imaging-derived lesions and biological measures. Pairwise deletion was used to handle missing values, and the two-tailed significance level was set at 0.05. To explore potential imaging markers of disease progression under SSA treatment, binary logistic regression models were constructed. Progression status was used as the dependent variable (0 = no progression, 1 = progression), while patient age, Ki67 index in metastatic sites, and quantitative imaging parameters (mean ROI values on DWI or ADC maps) were the independent predictors. Variable entry was accomplished by the enter method, and odds ratios (Exp(B)) with 95% confidence intervals were applied. All tests were two-tailed, and a p-value of less than 0.05 was considered statistically significant. All statistical analyses were conducted using IBM SPSS Statistics (version 29.0, IBM Corp., Armonk, NY, USA).

## Results

### Population characteristics

The final study population included a total of 17 patients. There were 6 females (35.3%) and 11 males (64.7%). The mean age of the patients was 57.2 years ± 12.6 (range, 32–80). Primary lesions were in the ileum (*n* = 11, 64.7%), pancreas (*n* = 6, 35.3%). Histologically, 2 patients (11.8%) had a G1 NET, 11 patients (64.7%) a G2 NET, and 4 patients (23.5%) had a G3 NET. All histologically analysed secondary lesions confirmed the same grade as the primary tumour.

For the treatment of metastatic disease, all patients received first-line therapy with SSAs. Disease progression during first-line therapy occurred in 11 patients (64.7%), who were subsequently treated with radioligand therapy (*n* = 7, 63.6%), chemotherapy (*n* = 3, 27.3%) or target therapy (*n* = 1, 9.1%). The mean interval between mpMRI and 68Ga-DOTA peptide PET-CT was 73 days (range, 12–176 days). Table 1 summarizes the patients’ characteristics.

**Table 1:** Patients’ characteristics

Patients’ characteristics		
Total of patients	17	
Mean age (range)	57.2 (32–80)	
Number (%)		
Sex		
Male	11 (64.7%)	
Female	6 (35.3%)	
Primary lesion localization		
Ileum	11 (64.7%)	
Pancreas	6 (35.3%)	
Grading		
G1	2 (11.8%)	
G2	11 (64.7%)	
G3	4 (23.5%)	
Functioning		
Yes	3 (17.6%)	
No	13 (76.5%)	
Unknown		1 (5.9%)
Therapy		
SSA	17 (100%)	
PRRT	7 (63.6%)	
Chemotherapy	3 (27.3%)	
Target therapy	1 (9.1%)	
Progression under SSA		
Yes	11 (64.7%)	
No	6 (35.3%)	

### Evaluation of the Diagnostic Role of DWI Compared to mpMRI and Functional Imaging

**Fig. 2:**
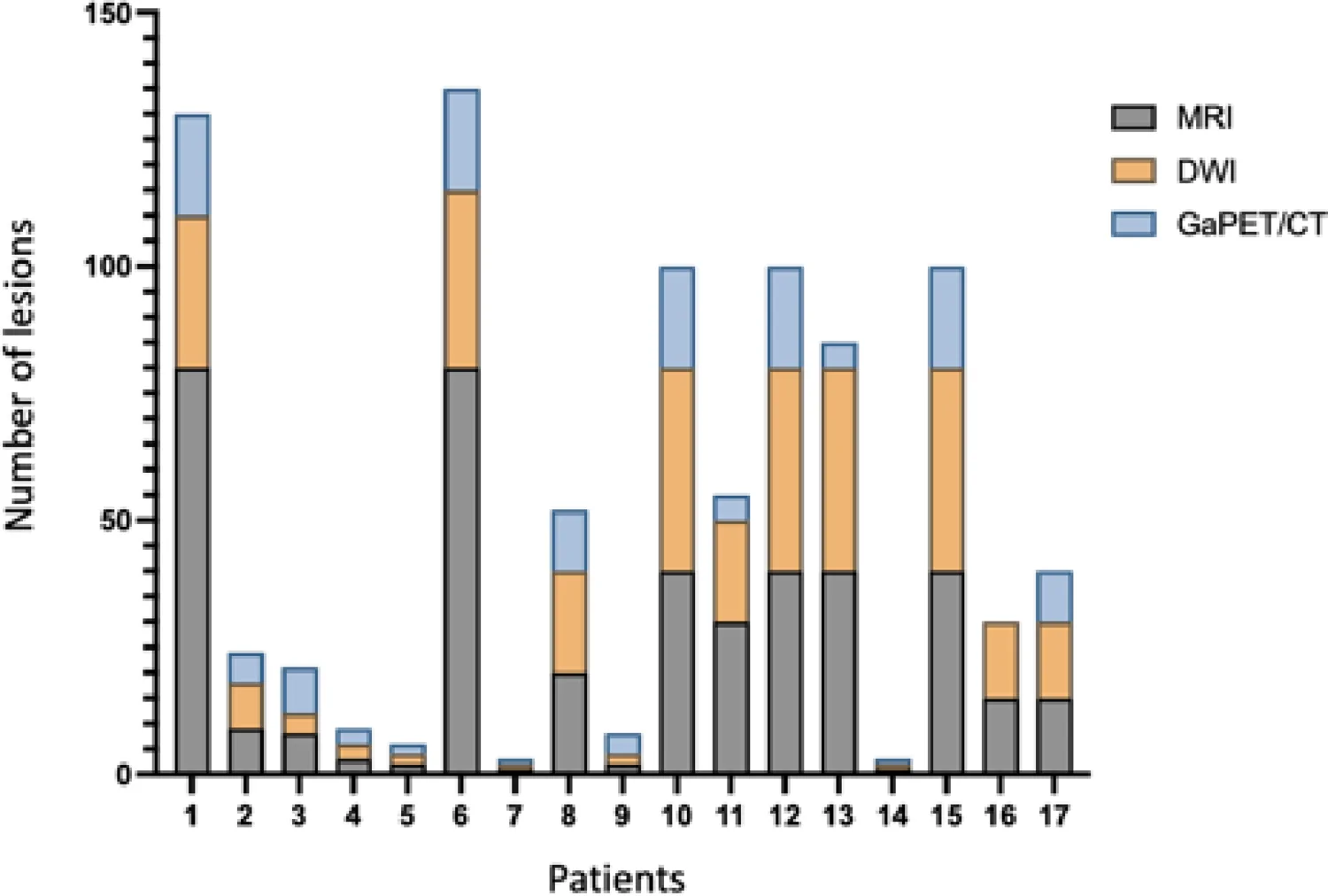
Number of lesions detected per individual patient

**Fig. 3:**
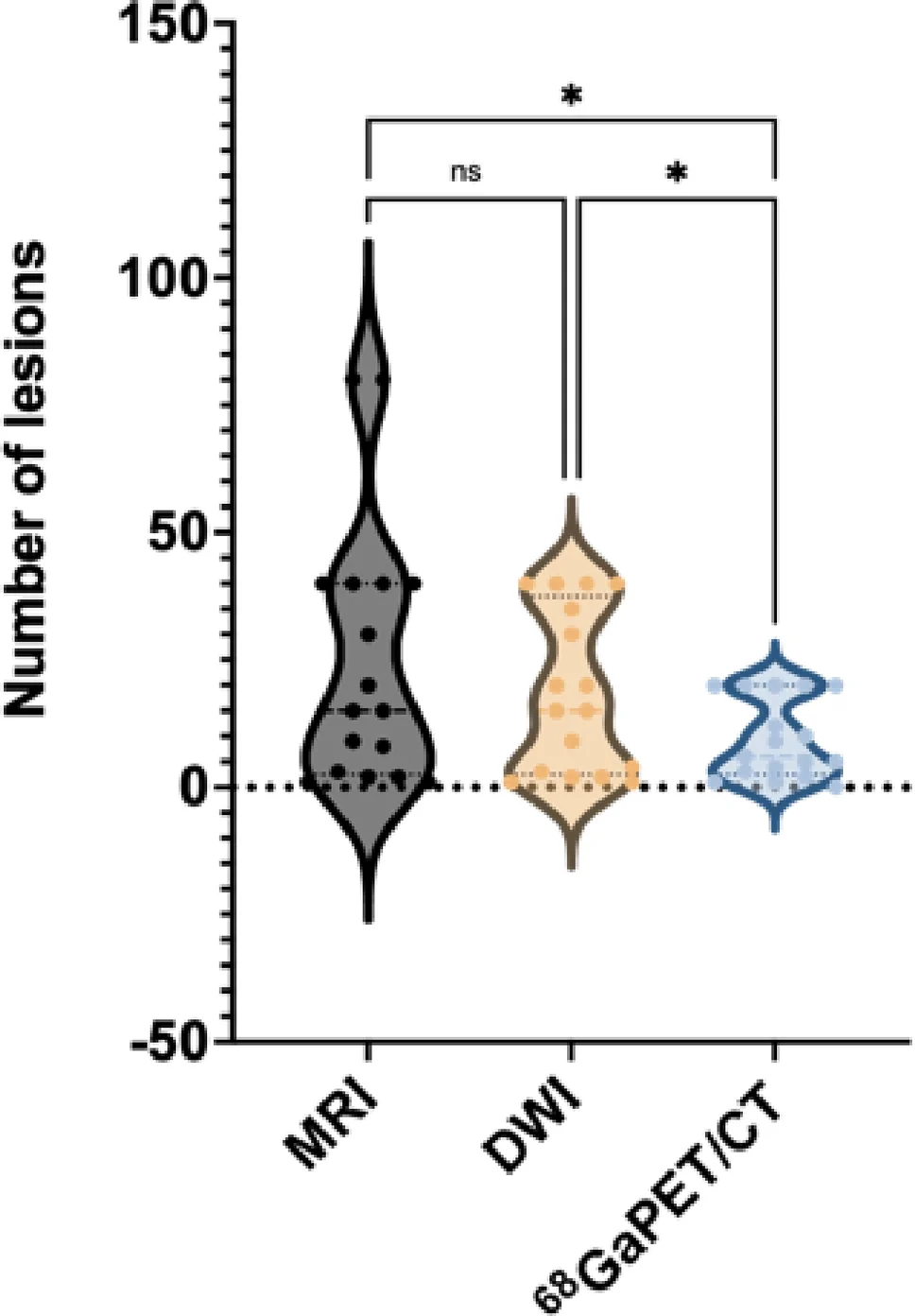
Comparison of lesion detection across imaging modalities

In line with the primary objective of comparing lesion detection across different imaging modalities using a lesion-based approach, the descriptive evaluation of lesion count revealed variability in detection performance between 

modalities. Multiparametric MRI, used as the reference imaging, detected a total of 426 lesions and a median of 15 (IQR 2.5–40) lesions per patient, DWI detected a similar median of 15 (IQR 2.5–36.5) lesions, while PET-CT revealed a reduced median of 6 (IQR 2.5–20) lesions (Fig. 2). Exploratory non-parametric comparison using Friedman’s test demonstrated a statistically significant difference in lesion counts across modalities (χ² = 12.047, df = 2, *p* = 0.002), with the mean rank highest for mpMRI (2.38), followed by DWI (2.15), and PET-CT (1.47), indicating higher detection performance for MRI-based modalities. Results were confirmed after excluding G3 patients (χ² = 10.414, df = 2, *p* = 0.005). Paired comparisons using Wilcoxon signed-rank tests showed higher lesion count for mpMRI compared with PET-CT (Z = − 2.975, *p* = 0.003) and for DWI compared with PET-CT (Z = − 2.873, *p* = 0.004), while differences between mpMRI and DWI did not reach statistical significance (Z = − 1.826, *p* = 0.068) (Fig. 3).

To account for intra-patient clustering of lesions, the Poisson GLMM was applied as a complementary analysis. Imaging modality was a significant fixed effect (Wald F = 56.77, *p* < 0.001), while the random intercept for patient was significant, indicating substantial inter-patient heterogeneity in lesion burden. Same results were obtained after the exclusion of G3 patients (Wald F = 44.45, *p* < 0.001). Estimated marginal means suggested higher lesion counts for mpMRI and DWI compared with PET-CT (mpMRI 14.85±14.25 vs. DWI 11.05±10.61 vs. PET-CT 5.51±5.29); however, after adjustment for multiple comparisons within the mixed-effects framework, pairwise contrasts did not retain statistical significance, likely reflecting the conservative nature of the model and the limited sample size. Comparison with a negative binomial mixed-effects model yielded higher AIC and BIC values, indicating no improvement in model fit and no strong evidence of overdispersion.

The analysis of agreement using Cohen’s Kappa between the different imaging modalities (MRI, DWI, and PET) could not be performed, as at least one of the variables in each pairwise comparison was constant across all observations, thereby preventing the computation of a meaningful concordance coefficient. The ICC for single measures was 0.517 (95% CI: 0.205–0.768), and the ICC for average measures was 0.762 (95% CI: 0.436–0.908), indicating moderate to good consistency of lesion counts across imaging modalities. Given the exploratory nature of the analysis and the distributional characteristics of the data, ICC estimates should be interpreted with caution.

**Fig. 4:**
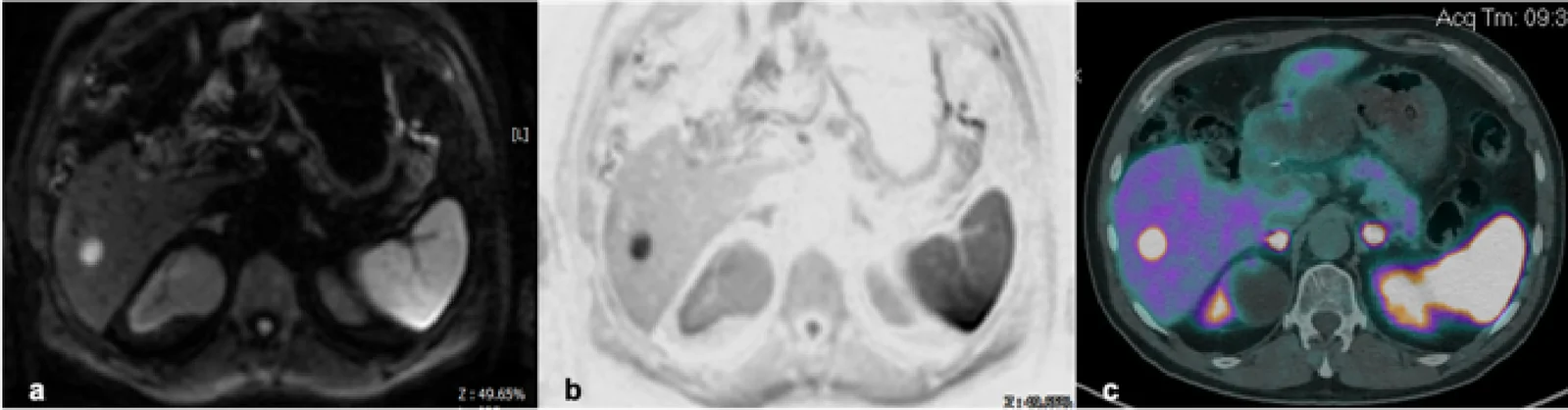
66-year-old patient with an ileal NET and a liver metastasis (segment V), clearly detected on the DWI image standard display (a), DWI inverted PET-like display (b), and 68Ga-DOTA peptide PET/CT (c)

**Fig. 5:**
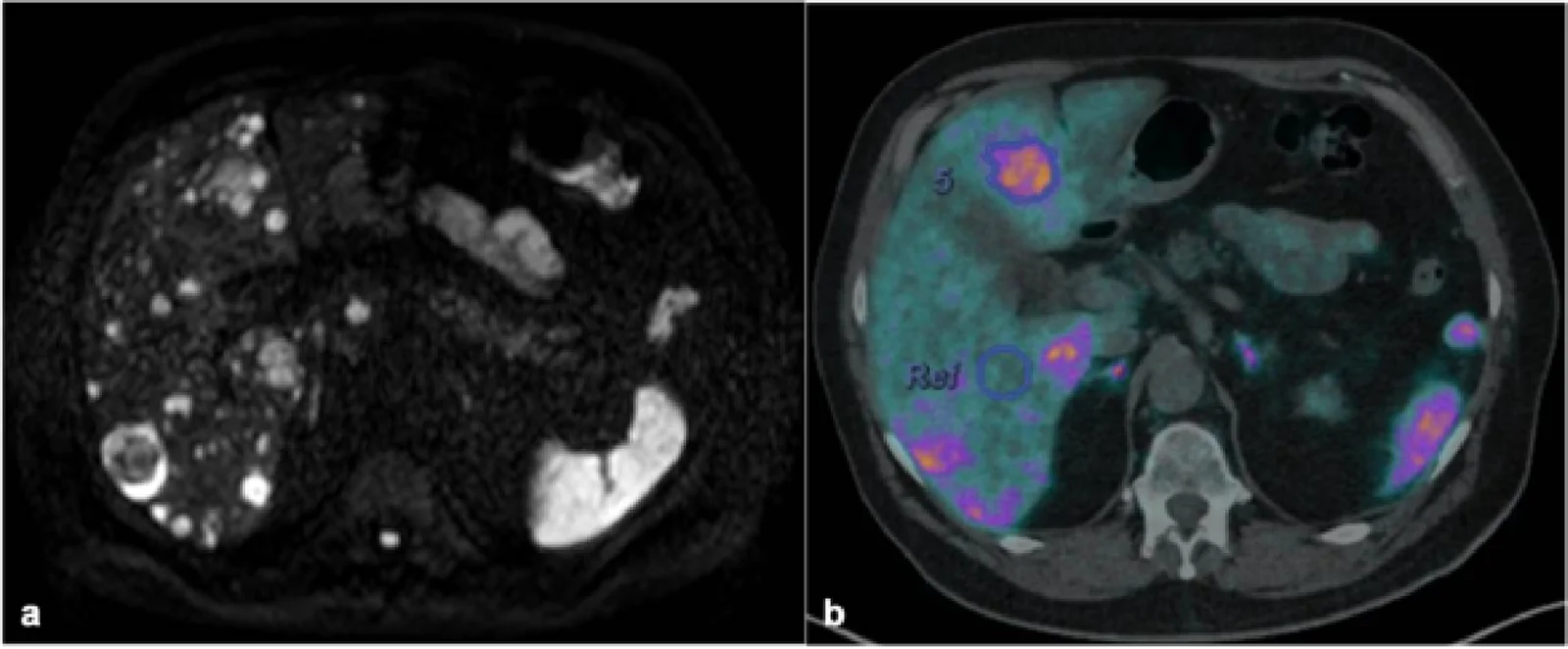
A 59-year-old patient affected by an ileal NET with multiple diffuse liver metastases of variable diameters, ranging from 0.5 to > 3 cm. Both smaller and larger lesions are clearly detected on the DWI image (a), whereas only larger lesions are detectable on the 68Ga-DOTA peptide PET-CT image(b)

Figure 4 shows an example of a lesion visualized by DWI and PET-CT, whereas Fig. 5 displays multiple LMs with widespread distribution detected only by DWI.

### Evaluation of the Prognostic Role of DWI Compared to Functional Imaging

In the enrolled population, a total of 37 target LMs were identified and analysed.

The mean lesion size was 25.43 ± 12.9 mm, ADC values were calculated for 29 lesions. The median ADC values (x10⁻³ mm²/s) with corresponding IQR were 0.679 (IQR 0.522–0.832). The median SUV values with corresponding IQR were 32.1 IQR (22.1–48.8).

Notably, lesion count by mpMRI correlated with Ki-67 of the primary tumor (*r* = 0.915, *p* = 0.011), and DWI lesion count showed a moderate correlation with Ki-67 (*r* = 0.862, *p* = 0.027), although the Ki-67 data were available for a limited subset.

To explore potential predictors of disease progression under SSA therapy, logistic regression models were constructed including age, Ki-67 of metastatic sites, and imaging-derived quantitative metrics. None of the models achieved full convergence due to the small sample size and low event rate. However, in the model including the ADC ROI for the largest lesion, the omnibus test of coefficients was significant (χ² = 10.431, df = 3, *p* = 0.015), and ADC showed an unstable and likely inflated odds ratio estimate (Exp(B) = 776.1), reflecting model instability due to sparse data and incomplete convergence. Therefore, these exploratory findings should be interpreted with extreme caution.

## Discussion

This monocentric, observational, both retrospective and prospective study suggests that DWI-MRI may detect a higher number of LMs than ^68^Ga-DOTA peptide PET-CT and shows performance comparable to mpMRI in terms of lesion burden assessment. The moderate-to-good consistency in lesion burden estimation across imaging modalities confirmed the reliability of lesion quantification across these imaging modalities. Significantly, correlation analysis showed putative biological correlations between tumour proliferation and liver disease burden, implicating MRI lesion load as a surrogate marker for disease aggressiveness. An exploratory prognostic role for ADC-based imaging biomarkers may be considered, although confirmation in larger prospective cohorts is warranted.

NENs display considerable heterogeneity in terms of anatomical site, histological subtypes, clinical manifestations, and biological aggressiveness. This variability results in the application of different therapeutic and follow-up strategies [23]. The main prognostic factors are tumor site, grade and stage. Notably, the presence of LMs has a major impact on both therapeutic management and patient prognosis, making their detection crucial and requiring frequent clinical and radiological assessments [22]. Moreover, due to the relatively favourable prognosis of patients with LMs from NETs, the duration of follow-up tends to be longer compared to patients with LMs from other primary tumours, thus increasing the total number of imaging evaluations performed [24].

Regarding morphological imaging, the superiority of mpMRI over CT in detecting LMs from NENs has been well established, and mpMRI is now considered the gold standard modality [22]. Recent literature particularly emphasizes the importance of the DWI sequence, which is now routinely included in all oncological MRI protocols. In the context of neuroendocrine disease, DWI has been shown to be more sensitive than both T2-weighted and contrast-enhanced dynamic sequences in identifying LMs [25].

A retrospective study by Lavelle et al. [26] analysed a cohort of 22 patients with LMs from NENs who underwent at least two mpMRI evaluations during follow-up. The authors compared lesion diameters measured in DWI versus the hepatobiliary phase sequence (used as the reference standard) and found a high level of agreement, leading them to conclude that DWI alone might be sufficient for the follow-up of these patients [26].

As for functional imaging, ^68^Ga-DOTA peptide PET-CT remains a valuable tool for detecting primary and metastatic well-differentiated NENs with good sensitivity and specificity. ^68^Ga-DOTA peptide PET-CT plays a pivotal role in the detection of occult primary NEN in metastatic patients, in the selection of candidates for radioligand therapy, and in the assessment of treatment response, allowing the identification of a possible metabolic response even in the presence of radiological stability. However, it has several limitations: false positives (e.g., inflammation, benign lesions, or physiological uptake), false negatives (e.g., sub-centimetric lesions, poorly differentiated tumours, or certain NET subtypes like insulinomas), lower diagnostic accuracy in cases of numerous lesions, risk of contrast medium allergy, and, albeit minimal, exposure to ionizing radiation [27, 28].

Given the higher spatial resolution of DWI-MRI compared to ^68^Ga-DOTA peptide PET-CT, we hypothesized that DWI may have greater sensitivity, especially in detecting sub-centimetric, multiple, or SSTR-low-expressing liver lesions. The results of our study confirmed this hypothesis and support the conclusions drawn by Lavelle et al. [26]. Our findings are also in line with those recently published by Haider et al., who conducted the only existing retrospective study aimed at directly comparing the diagnostic performance of DWI-MRI and 68Ga-DOTA peptide PET-CT in LMs from NETs. In their cohort of 32 patients and only 90 LMs, DWI-MRI showed a higher detection rate than PET-CT [29].

Accordingly, our study, including both prospective and retrospective phases and with a larger cohort of LMs (*n* = 426), validates the concept that, for the exclusive purpose of assessment of hepatic disease burden, follow-up of patients with LMs from NETs can be effectively done with the assistance of DWI alone, ideally augmented by a morphological sequence such as T2-weighted imaging. However, DWI is sensitive but not specific, because some benign liver lesions can show restricted diffusion [30]. Therefore, when lesions are not typical for NET metastases, additional mpMRI sequences (dynamic contrast or hepatobiliary phase imaging) or follow-up imaging are required for reliable lesion characterisation. Moreover, while mpMRI and DWI showed a tendency toward higher lesion detection compared with PET-CT, this effect was not consistent across patients suggesting that patient-level disease burden represents a relevant driver of lesion detectability rather than imaging technique alone.

The clinical usefulness of DWI lies in the intrinsic benefits of DWI-MRI, it is rapid, non-invasive, free of ionizing radiation and contrast agent . Replacement of mpMRI or PET-CT with DWI may result in cost reduction, time saving, and reduced waiting times, and more ready access to the appropriate imaging for patients, but these workflow advantages should be demonstrated in future prospective studies.

In addition to good diagnostic performance, the ideal imaging technique for patients with LMs from NETs should also allow prognostic stratification. Several recent publications have suggested a correlation between ADC values from DWI-MRI and tumour cellularity, indicating a possible role of ADC in assessing proliferative activity and thus tumour aggressiveness [7, 16]. To date, only one study has evaluated this correlation in patients with LMs from NENs, showing lower ADC values in G3 lesions compared to G1 and G2 [18]. In our study, mpMRI and DWI lesion count correlated with Ki67 levels at the primary site, although no such correlation was observed for metastatic Ki67. These findings suggest that radiologically apparent disease volume may reflect underlying tumour proliferation activity, at least at the primary site. The absence of correlation with secondary Ki67 can be seen because of biological heterogeneity between metastatic and primary lesions, a widely reported phenomenon in NEN biology [23]. Multivariate logistic regression models were used to explore possible imaging biomarkers of disease progression with SSA therapy. While DWI and ADC values were added to models to propose possible associations with progression status, none reached statistical significance or good convergence due to limited sample size and model instability. At the same time, it must be acknowledged that DWI techniques lack standardization, and ADC measurements are subject to significant inter-operator and inter-scanner variability [31, 32].

Taken together, these results emphasize the complementary diagnostic and potentially prognostic value of combining structural mpMRI, DWI-MRI, and ^68^Ga-DOTA peptides PET-CT imaging in the management of NENs. The significant correlation between lesion burden and Ki67 at the primary site may support the inclusion of radiologic metrics as surrogate markers of tumour proliferation in early-stage assessment. Furthermore, the consistency of ROI-based quantification supports its clinical utility in both routine practice and research protocols.

However, the present study has some limitations. First, the small sample size, particularly for the prospective phase, limits generalizability and statistical power of the findings, which need confirmation in further larger prospective studies. Second, the use of mpMRI as a surrogate reference standard, with partial incorporation of DWI findings in equivocal cases may have introduced incorporation bias and potentially overestimated the diagnostic performance of DWI. However, DWI was not used as an isolated determinant of lesion malignancy, in fact lesion classification required concordance across multiple MRI sequences, functional imaging, and all available clinical information, reflecting common clinical practice. Third, in cases of extensive metastatic burden, lesion counts were derived from a standardized regional sampling approach rather than complete lesion enumeration, which may influence absolute lesion counts but was applied consistently across imaging modalities and performed by experienced radiologists in consensus using predefined criteria. Fourth, the convergence issues with logistic regression highlight the need for careful modelling strategies, particularly when the outcome is rare, or the predictor variables exhibit low variability. Finally, the use of both lesion- and patient-level analytical approaches, although clustering was addressed using mixed-effects modelling, may introduce a degree of heterogeneity in the methodological framework. However, this complementary strategy allows a more comprehensive evaluation by accounting for both lesion-level and patient-level variability, which could represent a strength of the study.

## Conclusion

In conclusion, our study suggests that DWI-MRI may detect a greater number of LMs from GEP-NETs compared to ^68^Ga-DOTA peptide PET-CT and showed similar lesion burden assessment to mpMRI, the current gold standard. These findings indicate that DWI-MRI may represent a promising alternative technique for the short-term follow-up of patients with LMs from GEP-NETs. If validated in larger prospective cohort, this approach may offer not only economic savings but, more importantly, greater time efficiency and improved accessibility to essential imaging studies for patients who require them. Although further comparative studies with larger patient cohorts are needed to assess its prognostic value, our work provides preliminary evidence supporting the potential prognostic role of diffusion and lesion-based imaging parameters.
